# A Comprehension- or a Production-Based Monitor? Response to Roelofs (2020)

**DOI:** 10.5334/joc.102

**Published:** 2020-09-03

**Authors:** Nazbanou Nozari

**Affiliations:** 1 Department of Psychology, Carnegie Mellon University, US; 2 Center for Neural Basis Cognition (CNBC), US

**Keywords:** Speech monitoring, conflict, perceptual loop, forward models

## Abstract

Roelofs (2020) has put forth a rebuttal of the criticisms raised against comprehension-based monitoring and has also raised a number of objections against production-based monitors. In this response, I clarify that the model defended by Roelofs is not a comprehension-based monitor, but belongs to a class of monitoring models which I refer to as production-perception models. I review comprehension-based and production-perception models, highlight the strength of each, and point out the differences between them. I then discuss the limitations of both for monitoring production at higher levels, which has been the motivation for production-based monitors. Next, I address the specific criticisms raised by Roelofs (2020) in light of the current evidence. I end by presenting several lines of arguments that preclude a single monitoring mechanism as meeting all the demands of monitoring in a task as complex as communication. A more fruitful avenue is perhaps to focus on what theories are compatible with the nature of representations at specific levels of the production system and with specific aims of monitoring in language production.

Roelofs (2020) has put forth a rebuttal of the criticisms raised against comprehension-based monitoring as the main monitoring mechanism in speech production. He has also raised a number of objections against production-based monitors. In this response, I clarify that the model defended by Roelofs is not a comprehension-based monitor, but an example of another class of monitoring models, which I refer to as production-perception models. I review comprehension-based and production-perception monitors and point out fundamental differences between them which prevent lumping them together as a single monitoring mechanism. In doing so, I stress the critical importance of each class for certain aspects of speech monitoring. I then discuss the limitations of these mechanisms for rapid constant monitoring of production at higher levels, and lay out the foundational arguments for a production-based monitor. Next, I address the specific criticisms raised by Roelofs (2020) in light of the current evidence. I end by emphasizing that the complexity of language (generative grammar), of the language production system (the multiple layers of representations), and of communication (tailoring utterances to an audience), together with the great differences in various monitoring aims (e.g., adjusting voice loudness, revising syntactic structure, more quickly choosing one of two words, etc.) simply precludes a single monitoring mechanism as meeting all the demands of monitoring. A more fruitful avenue might be to focus on what theories are compatible with the nature of representations at certain levels of the production system and with specific aims of monitoring in language production.

## Comprehension-based monitoring

Comprehension-based accounts comprise a classic group of monitoring models which share the premise that comprehension is the basis for monitoring in production. Although discussed in various forms since the 60s (e.g., Garrett, 1980; Laver, 1973), they were accepted as a formal theory only after Levelt’s (1983) proposal of the perceptual loop account. The account is summarized by Levelt as the following: *“self-produced inner or overt speech is perceived, parsed and checked with respect to intentional and contextual appropriateness, agreement of intended and delivered message, and linguistic correctness. When trouble is detected, central corrective action is taken.”* (Levelt, 1983, p. 50). This concise definition conveys several key points regarding the perceptual loop account and comprehension-based monitors in general. Note the critical use of the terms “parsing”, “intentional appropriateness” and “intended message” repeatedly found in Levelt’s writings on the functioning of the perceptual loop, which points to a mechanism that a) is attentional, b) has access to a large variety of information, including knowledge about phonology and syntax, and c) is used for detecting errors in one’s own speech as well as in other people’s speech. The intentional nature of this monitoring mechanism is further emphasized by Levelt (1983; pp. 96–97): *“The great advantage of a perceptual theory is that controlling one’s own speech is like* ***attending*** *to somebody else’s talk. This makes it natural for the speaker to apply the same parsing procedures and sources of knowledge to his own speech as to other people’s speech. More particularly, the speaker will **try and interpret** his own speech in the context of what was previously said by himself or by another person. He may thus become aware of ambiguity, vagueness, indeterminacy of reference, incoherence, etc.”*.

Therein lies the strength and elegance of the comprehension-based account: the monitoring “device”, i.e., the parser, is identical for self- and other-produced speech. Moreover, in both cases, the parser only receives the end-product of the production processes (overt speech or inner speech), and compares it to a standard, i.e., the target. The only difference is the nature of the target, the intended message in one’s own speech, and the discourse model in other people’s speech (Levelt, 1983, p. 97). It is important to note that this theoretical view, unlike the production-perception models reviewed later, does not require structural connections between representations in production and perception within the speaker, as such connections are obviously absent between the perceptual system of a listener and the production system of another speaker. Since inner speech can be “listened to” similarly to overt speech, it should be theoretically possible for the parser to receive its input in a way similar to how it receives the speech of others, without direct connections between the two sets of representations. Such a position is quite reasonable, and the resulting model would be truly parsimonious in positing a single mechanism for monitoring self- and other-produced speech. The theory also makes an explicit claim that conscious awareness of errors is a necessary prerequisite for the initiation of any corrective actions (Levelt, 1983, p. 45). The account is also remarkable in its scope: since listeners try to extract meaning out of all aspects of an utterance (from speaker’s intentions to speech sounds), a comprehension-based monitor that operates in a similar manner, i.e., by trying to “listen” to the speaker’s internal speech, should also be capable of monitoring all aspects of communication.

In a nutshell, comprehension-based monitoring entails conscious and deliberate usage of the parser to compare the output of speech production, from self or others, to some standard, and to decide on the corrective action (if any) to be taken, based on the speaker’s knowledge. The identical nature of the parser’s operation on overt and inner speech, without any further assumptions, confers to the theory its great advantage, which “[…] is that controlling one’s own speech is like attending to somebody else’s talk.” (Levelt 1983; p. 96–97). Moreover, its attentional and deliberate nature easily expands its scope to monitoring for complex aspects of communication, such as tailoring the utterance simultaneously to speaker’s intentions and interlocuter’s background knowledge, discourse context, and common ground.

## Production-perception models of monitoring

Under this group are models in which perceptual consequences of motor actions are anticipated and used to adjust motor actions in order to achieve the desired outcome. I avoid the term *internal models*, because it has been used in the literature to refer to forward models, inverse models (defined below), or a combination of the two, and is thus ambiguous. I instead opt for the unambiguous term “production-perception” monitoring models, to emphasize that the monitoring mechanisms proposed by these models hinge on the interaction between production and perception. On the perceptual side, these models use both acoustic and somatosensory representations, the former of which is also involved in comprehension-based monitoring. The nature of the comparative process is, however, very different between comprehension-based and production-perception models. Comparisons in the former class are conscious and informed by linguistic knowledge, whereas comparisons in the latter (as well as the subsequent adjustments) are much less conscious and influenced by explicit knowledge. This is, to a great extent, due to the scope of the production-perception models, which is primarily motor control of speech. Two such models have been proposed for speech monitoring, which have nontrivial differences in their mechanisms of operation. Below, I give a brief overview of both.

**Figure 1:**
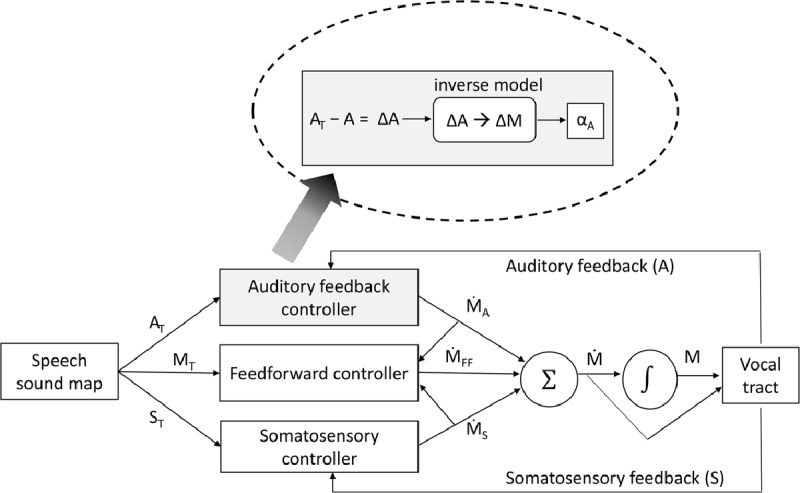
Schematic of the DIVA model (adapted from Guenther, 2016). The inset shows the calculations within the auditory feedback controller. A_T_ = Auditory Target; M_T_ = Motor Target; S_T_ = Somatosensory Target; Ṁ_A_ and Ṁ_S_ = corrective movement commands from auditory and sensory routes, respectively; Ṁ_FF_ = feedforward movement command; Ṁ = overall movement command. M = motor position command; α_A_ = gain factor.

The most detailed, well tested, and neurally plausible production-perception model of speech motor control is “directions into velocities of articulators” model, or DIVA (Guenther, 1994), and its neurally implemented version, gradient order DIVA model, or GODIVA (Bohland, Bullock, & Guenther, 2010). Figure 1 shows a schematic of this model adapted from Guenther (2016). Each attempt at producing a word starts with the activation of the speech sound map. This, in turn, activates three signals: an auditory target (A_T_), a somatosensory target (S_T_), and a stored motor program (M_T_). Note that the activation of auditory and somatosensory representations is anticipated before the output of motor action actually activates such representations. This is called a *forward model*. The role of the controllers is to compare the desired states of the system (A_T_, S_T_, and M_T_) with its actual states. Once the output of the motor action is available, perceptual targets A_T_ and S_T_ are compared against that output, i.e., the auditory and somatosensory feedback. The motor target M_T_ is also compared against the actual motor state (not shown in the figure). In all cases, a mismatch between the desired and actual states of the system generates *corrective movements commands* (M with the over-dot, which represents the time derivative). The sum of the three corrective movement commands (Ṁ_A_, Ṁ_S_, and Ṁ_FF_) produces the overall movement command Ṁ, which, along with its integration over time as the motor position code M, is sent to the vocal tract. Perceptual corrective motor commands Ṁ_A_ and Ṁ_S_ will also update the motor target M_T_ for future production.

Although perceptual representations are involved in monitoring and regulation of production, the comparison is always between perceptual targets and perceptual outcomes, and not between production targets and perceptual outcomes directly. This can be seen in the blown-up inset in Figure 1. The mismatch is calculated between the auditory target (A_T_) and the auditory outcome (A). The resulting ΔA is thus in perceptual space. For ΔA to influence production it must be translated into a ΔM in production space, an operation that is carried out by an *inverse model*. Scaled by a gain factor (α_A_), the resulting Ṁ_A_ represents the corrective movement command. Similar computations are carried out in the somatosensory feedback loop. DIVA explains how speakers adjust their motor production based on auditory and somatosensory perturbations and provides a mechanistic account for how children learn to produce the sounds of the language they hear. While the model was not designed to explain the detection of errors before they become overt, early detection should be possible in cases where the proprioceptive feedback from the very onset of the word is incompatible with the anticipated somatosensory target. The model, however, does not explain detection of errors before any motor act has been initiated.

**Figure 2:**
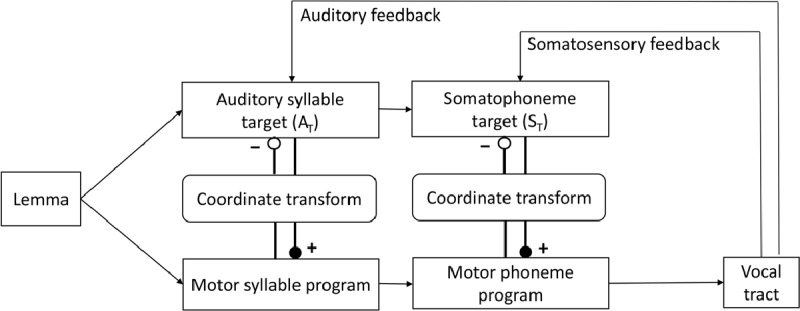
Schematic of the HSFC model (adapted from Hickok, 2012). The internal monitoring loop operates via connections between motor programs and perceptual targets (A_T_ and S_T_) mediated by the coordinate transform system. Note the direction of the connections in bold: connections from perceptual to motor representations are excitatory (filled circles), while the connections from motor to perceptual representations are inhibitory (empty circles).

The second production-perception-based model is the “hierarchical state feedback control” model, or HSFC (Hickok, 2012). A schematic of this model is shown in Figure 2. Similar to DIVA, the model has two sources of perceptual feedback, auditory and somatosensory, which are compared to auditory target (A_T_) and somatosensory target (S_T_), respectively. Also like DIVA, the cross-talk between representations in production and perception space requires translation through the coordinate transform system. HSFC is different from DIVA in two ways: First, it proposes a hierarchy of representations with phonemes under syllables, and links them to somatosensory and acoustic representations, respectively. Second, in order to model the internal channel of monitoring, HSFC proposes an internal link between representations on the production and perception sides. Internal monitoring is achieved in the following way: Activation of an abstract word form (lemma) through its semantic features activates both production and perceptual representations (first syllable-level representations, then phoneme-level representations). Perceptual representations contribute further to the activation of production representations (excitatory connections in Figure 2). Production representations, on the other hand, suppress the activation of their corresponding perceptual representation (inhibitory connections in Figure 2). If all goes well, as the production representation gains more activation it drives down the activation of the corresponding perceptual representation back towards its baseline. In errors, the lemma activates the correct perceptual representation but the incorrect production representation. The perceptual representation thus remains active due to the absence of enforced inhibition. This persistent activation is translated into an error signal.

In summary, production-perception models utilize various sources of perceptual feedback, most importantly acoustic and somatosensory, and compare them against the predicted sensory consequences of motor actions. In the case of HSFC this is supplemented by an internal loop, within which corresponding representations in production and perception regulate each other. Unlike comprehension-based models, no deliberate parsing or explicit knowledge is required or utilized in the monitoring process.

## Production-based monitoring

Comprehension-based models draw on linguistic and discourse knowledge in composing well-formed and informative utterances that are best tailored to the current audience. Perception-production models are ideal models of motor control of speech. Why then do we need production monitoring? Answering this question is important in order to understand that production monitoring is not “in competition” with comprehension-based or production-perception monitoring models, because it covers parts of the process that are not easily covered by the other two classes of models. The answer goes back to the goal of monitoring. While sometimes equated with “error detection”, monitoring serves the much larger role of regulating of the production system. This view is not new; Levelt (1983) deemed monitoring necessary in order “to create instructions for adjustment” (p. 50). The multitude of studies now available on the role of inhibitory control in language production (see Nozari, 2018, for a review), also point to the necessity of a mechanism that determines the need for the recruitment of control, in order to optimize production performance.

With adjustment of performance as the goal of monitoring in mind, Levelt (1983) examined two alternatives, production- and comprehension-based accounts. The production-based account he evaluated at the time was the one proposed by Laver (1980). In Laver’s account, each level of processing in production is equipped with a mini-monitor which has access to the target of that level, and compares that target to the outcome of processing from that level. Levelt (1983) raised two main criticisms against Laver’s model: a) Speakers do not have access to the intermediate levels of production, thus conscious comparisons within layers seems farfetched. b) Since these mini-monitors were additions to the production system with the sole purpose of inspecting each layer’s output, Levelt argued that the production-based account was unparsimonious compared to a comprehension-based account which simply utilized the parser.

Both criticisms are on point. A third criticism, which plays a key role in evaluating any monitoring model, is unnecessary duplication of knowledge (MacKay, 1987; Nozari & Novick, 2017). In his production-based monitoring theory, Laver (1980) assumes that the target of production is available within the same layer of production in which the response is being generated. The question then is, if the target is already available in the same format as the response, why is it not produced in the first place? Together, these objections provide a good case against Laver-type production monitoring. The same criticisms push towards a model a) that only has access to the final output of production, b) within which comparisons take deliberation and effort, hence the possibility of delayed interruptions, and c) in which two sets of distinct representations are available without violating assumptions of parsimony. Levelt’s perceptual loop was thus a carefully constructed proposal to address these issues, and it did so splendidly.

The problems with the old production-based monitors and the emergence of the perceptual loop theory, however, naturally limited monitoring to the end product of production, i.e., segments (syllables or phonemes depending on the specific theory). Therefore, important parts of the production process, e.g., lexical selection or abstract construction of syntactic trees, were left outside of the scope of monitoring. There are two possible solutions here: 1) To accept that this is truly the case, and the monitor only has access to the final outcome of production. 2) To assume that the information generated in other stages of production is useful to the monitor and is employed to control and regulate production at different stages. The current evidence speaks against the former possibility: data from individuals with aphasia have shown dissociations in the individuals’ abilities to monitor semantic and phonological errors (e.g., Nozari, Dell, & Schwartz, 2011; Stark, 1988). Schuchard, Middleton, and Schwartz (2017) showed further differences in how errors were treated: while repair attempts were more common for phonological than semantic errors, negations (i.e., rejecting a response without providing a repair) were much more prevalent for semantic (38%) than phonological (8%) errors. Interestingly, negations were also significantly faster than repairs. These data suggest that where in the production system the error arises matters for how it is detected and subsequently treated. Negations are particularly telling: a response, usually a lexical item, can be rejected without there being any clear target to replace it. I will return to this issue later. The critical point relevant to the current discussion is that monitoring is unlikely to be limited to the outcome of the production process.

The premise that monitoring happens at multiple stages of production, which I call *multi-level monitoring*, is in fact quite popular. For example, Hickok (2012) proposes a hierarchical monitoring system with two levels of monitoring: monitoring at the level of what he refers to as “phonemes” (but may be better aligned with articulatory features) is carried out by the somatosensory system, which controls the vocal tract trajectories, whereas monitoring at the level of “syllables” is carried out by the auditory system which provides higher level control. This is a great example of applying the logic of motor control to multiple levels of the language production system in order to connect the motor speech and psycholinguistic traditions. His extension of monitoring to the psycholinguistic realm did not—and could not—go far beyond phonology, because of his view “that speech production is fundamentally a motor control problem” (Hickok, 2012; p. 137), a view that is diametrically opposed to the psycholinguistic view, which sees motor control as only one part of a much larger dilemma that speakers face when producing language. This is not a bad thing at all. It simply means that the model focuses on a certain part of the production system and deals very carefully with the intricacies of operations in that part. The advantage of limiting the scope of the extension of a motor theory to one level of abstraction above motor control was, however, that the theory remained plausible and applicable. There is some evidence that input (perceptual) and output (production) phonology may be separable (e.g., Howard & Nickels, 2005; Jacquemot, Dupoux, & Bachoud-Lévi, 2007), thus the basic premise of a production-perception based model, i.e., comparison between two separate sets of representations, is reasonable at the level of phonology. Other sympathizers with the multi-level monitoring, e.g., Pickering and Garrod (2013), have claimed extensions of similar production-perception models to *all* layers of production, in order to explain detection of lexical, syntactic, and other kinds of errors. The problem with this extension to levels higher than phonology is that the basic requirements of a perception-production model are no longer met at these layers. I will refer to this as the problem of *duplicate representation*.

### The problem of duplicate representations and its solution: non-comparative monitoring

Perception-production models like Hickok’s (2012) model have an important requirement: there need to be *two separate sets of representations*, one on the production side and one on the perception side, for comparisons to be possible. This requirement is readily met in lower parts of the speech production system; motor representations are clearly separate from auditory and somatosensory representations. This level of production thus easily lends itself to the application of perception-based models. Applying similar models to higher levels, e.g., lemmas, requires that there be two separate sets of lemmas, one on the production side, one on the perception side. The same is true for syntactic frames, etc. To my knowledge, there is no evidence, behavioral or neural, to suggest that such a separation exists at these levels. Without separate representations at that level, there are two options left: a) delaying monitoring by a production-perception monitor until the signal reaches levels where duplicate representations do exist and then carry out the comparisons, or b) to propose a different monitoring mechanism which is compatible with the nature of representations at this level. The drawbacks of the former option have been discussed above. The latter option is the idea behind a production-based monitor.

The absence of duplicate representations makes comparison with an external standard impossible, i.e., there is no target external to the production system to which a response can be compared. Interestingly, however, data suggest that a target may not be necessary to detect an error. In earlier sections, I mentioned “negation” as a common manifestation of monitoring in individuals with aphasia; the speaker knows that their answer is incorrect but does not know what the correct answer is. This finding implies that the system may be able to rely on heuristics to estimate the probability of an error in the absence of a standard target, similar to how a smoke detector can, under most circumstances, reliably signal the probability of a fire by detecting the level of smoke, without having any explicit knowledge of what a fire is. The first formulation of the idea as a monitoring process was MacKay’s node structure theory (MacKay, 1992), where he proposed that a system which routinely falls into familiar patterns may be able to recognize unfamiliar patterns as outliers. Our conflict-based account was one way to implement the recognition of an unfamiliar pattern (Nozari et al., 2011).

In a nutshell, conflict monitoring (Botvinick, Braver, Barch, Carter, & Cohen, 2001) is based on the idea that, in trouble-free situations, one representation (usually the correct response) has much higher activation than the others. When several representations have similar levels of activation, it is a sign that trouble is brewing; there is no longer a clear response in sight. The system can thus use this information to “guess” the probability of an error. The closer the activations of the items, captured in the notion of *conflict*, the higher the likelihood of generating an error. Conflict arises from the natural dynamics of the production system, e.g., mapping semantic representations to lexical items, which we have modeled using a neural network (Nozari et al., 2011). A decision about how much conflict is high enough to be detected as an error is a task for a decision making framework (see Nozari & Hepner, 2018, under review, and the accompanying commentaries for the proposed application of signal detection theory as the decision making framework). The model thus uses the information generated during primary production processes at each level of the production system to gauge the probability of an error. Since this probability can be decreased by applying appropriate control, or by simply delaying production until the mapping processes have converged more closely on a certain representation, the conflict-based monitor can also allow moment-by-moment adjustments to optimize performance based on goals. For example, when competition at the lexical level is high, the conflict signal can prolong the selection process until conflict falls below a certain level (i.e., competitive selection, e.g., Roelofs, 2004). If, however, speed is favored over accuracy, a similar framework can explain how a non-competitive selection profile (e.g., Mahon, Costa, Peterson, Vargas, & Caramazza, 2007) can arise, simply by adjusting the criterion for how much conflict is acceptable (Nozari & Hepner, 2019a, b).

In short, the conflict-based model achieves the goal of task adjustments defined by Levelt (1983), without suffering from the criticisms he raised against Laver-type production-based models. It does not require redundant representations, it does not duplicate knowledge at each layer of the production system, and it does not need conscious access to those layers, and yet it can monitor information at levels where perception-based monitors are not applicable.

Let me point out that there is a special way to implement comparative monitoring using the same set of representations: by appealing to temporal differences. The “target” in this case is not external and deterministic like DIVA and other models that use the actual perceptual (auditory or proprioceptive) signal. It is an “estimated target”, meaning that the activation of a representation at an earlier time is taken as a probabilistic standard to be compared to itself at a later point in time. Two variants of such models are possible:

- A *feedforward temporal model*. A model that compares the activation of a given representation (or representations) to the activation of the same representation(s) at different points in time as activation continues to spread in the system and the signal-to-noise ratio increases. For example, the activation of lexical items “cat” and “dog” are measured once at time t_1_ and once at a later time point, t_2_. If the general pattern is similar in both time points (e.g., “cat” is consistently more activated than “dog”), the system proceeds to generate an output. If, on the other hand, the pattern has substantially changed from t_1_ to t_2_ (e.g., “cat” was more activated than “dog” at t_1_ but is less activated than “dog” at t_2_) the system detects a discrepancy (a conflict between the two states), and generates an error signal. Another way of looking at this is to say that the model “predicts” a certain state through a first-pass neural sweep at t_1_, and then “verifies” that states through full neural activation at t_2_. This is plausible, and does not require duplicate representations, but note the critical point: this comparison happens *within the same system*, not across production and comprehension systems. For representations such as lexical items that are shared between production and comprehension (i.e., one set of representations), such a model boils down to a conflict-based model with a temporal component. A similar framework has been proposed for applying fast and automatic repairs (Nooteboom & Quené, 2020; Nozari, Martin, & McCloskey, 2019).
- A *feedback temporal model*. Similar to the feedforward temporal model, comparisons in this model are time-dependent, but rely on feedback from lower levels. In an interactive production model, activation from lexical items spreads to phonemes, and activated phonemes feed back to the lexical items they are connected to. If the activation that is sent forward is not fed back to the same nodes, a discrepancy is detected (see Postma & Kolk, 1993, for a similar proposal). This is also a perfectly plausible model, but it is a production-based monitor operating in an inherently interactive production system (e.g., Dell, 1986; Rapp & Goldrick, 2000). It is not impossible to imagine that activation, instead of directly feeding back from phonemes to lexical items, would travel all the way to perceptual representations, and then back to the same lexical items (see below for a similar proposal in Roelofs, this issue), and hence involved the perceptual system. But one must wonder why such a delay is necessary or plausible given the strong empirical evidence in favor of feedback within the production system in all production modalities (Dell, 1986; Pinet & Nozari, 2018; Rapp & Goldrick, 2000).

## Roelofs’s model

Roelofs abandons the notion of conscious and attentional monitoring which gives comprehension-based monitors their strength and parsimony in adopting a similar mechanism in monitoring one’s own speech and other people’s speech and covering a wide range of monitoring functions. Thus, his model does not fit the description of a comprehension-based monitor. It is rather a production-perception monitor. In terms of its general architecture, the model is closest to Hickok’s (2012) model in positing internal links between representations on the production and perception sides. The model differs from Hickok’s model in three important ways:

1. Although it connects production and perceptual representations directly, the monitoring mechanism is fundamentally different. In Hickok’s model, as explained earlier, production representations automatically suppress perceptual representations. This is motivated by neural evidence of the suppression of the auditory cortex during speaking (Aliu, Houde, & Nagarajan, 2008; Christoffels, Ven, Waldorp, Formisano, & Schiller, 2011). Roelofs, instead, proposes “verification operations by means of condition-action production rules.” (Roelofs, 2020; 10). The obvious question here is why resort to condition-action rules? This criticism is not new. In their commentary on Levelt et al. (1999), Santiago and MacKay criticized the use of such verification processes as sophisticated homunculi, and the proposal of different production rules at different planning levels put forth by Roelofs (2020) exacerbates this problem. Their criticism is apt: if monitoring can be achieved without any such verification processes (and the alternative models of monitoring show that it can) proposing such mechanisms for monitoring is hard to justify.
  But there is an even bigger problem here: the nature of these condition-action rules. In his review of the current paper, Roelofs clarified this by pointing out that in his view, monitoring is one of the goals of speaking (in addition to the communication goal) and suggested that condition-action rules are enabled by such a goal. If this is indeed the claim, then one must argue that monitoring performance is never the “goal” of any action. Speakers do not speak with the goal of detecting their errors; they speak with the goal of communicating a message. Considering the general redundancy of condition-action goals for the purpose of monitoring and the specific problem in defining the nature of such rules, one must wonder why adopting them is desirable in the first place. Note that Kröger et al. (2016), which Roelofs cites as support for the use of condition-action rules, use such rules in a very different capacity, i.e., to decide how the system *acts* (e.g., speak, halt, etc.) in different conditions. For example, the action “halt” is selected if the difference between semantic pointers activated in production and perception routes is small at multiple layers in the system. Thus the information required for monitoring does not come from condition-action rules, the behavior that follows monitoring is guided but such rules.
2. The second difference between Roelofs’s model and Hickok’s HSFC is that the latter, similar to DIVA, proposes a translation process (“coordinate transform”) to link perceptual representations with those on the production side. This proposal has a computational motivation (different nature of production and perception representations), as well as neural support (Hickok, Buchsbaum, Humphries, & Muftuler, 2003; Hickok, Houde, & Rong, 2011; Hickok, Okada, & Serences, 2009; Houde & Nagarajan, 2011), and has important implications for predicting the neural regions involved in monitoring. In line with this neurobiological plausibility, HSFC, similar to other production-perception models, posits multiple sources of monitoring, including both acoustic and somatosensory representations. This is important both for predicting the neural regions involved in monitoring, as well as for a commitment to a multiple-channel view of monitoring. Roelofs’s model does not have these components.
3. Finally, the level at which monitoring is done appears to be different between the two models. HSFC restricts monitoring to post-lexical levels, whereas Roelofs extends monitoring to higher levels of production. As explained earlier, under “the problem of duplicate representations”, a direct extension of forward and inverse models to higher levels in the language production system runs into the problem of duplicate representations. Since there is no evidence —that I know of— that validate the existence of two sets of lexical items, this solution is out. Roelofs’s proposed mechanism is closest to the *feedback temporal model*, but with a much more prolonged pathway: instead of lemmas receiving feedback from phonemes that they activate within the production system, they must wait for activation to propagate from the production to the perceptual system, and then be fed back to lemmas through the latter. There are at least two mechanisms that would make a feedback temporal model much faster: a) production-based monitor with feedback, the evidence for which is plenty, or b) a model like Hickok’s (2012) that assumes quick activation of both perceptual and production representations directly from their link to lexical items (not through a serial loop of lemma → output phonology → input phonology → lemma), and is much more compatible with data suggesting rapid ignition in the language system at the beginning of a production attempt (e.g., Strijkers & Costa, 2016).

To summarize, Roelofs’s proposed model lacks a key feature of comprehension-based monitors, i.e., conscious and deliberate processing. In that sense it is closer to production-perception and production-based monitors. Thus, this model is not suitable for addressing the criticisms raised against comprehension-based monitors. Apart from that debate, as a distinct account of monitoring, its mechanisms seem to be less efficient than those proposed by the current production-perception or production-based monitoring accounts. It thus needs to be justified why the model should be adopted over alternative proposals.

## Roelofs’s criticisms and the empirical evidence

Roelofs (2020) addressed several criticisms against comprehension-based monitors, and at the same time raised his own objections against production-based monitors. In this section, I briefly respond to these objections.

### The cross-talk problem

Raised originally by Vigliocco & Hartsuiker (2002), the cross-talk problem points out the temporal discrepancy between sound streams in inner and overt speech, with the latter being slightly delayed due to articulatory buffering. The question then is how a monitor which uses the same information in the comprehension system distinguishes between the two. Roelofs’s proposed solution to this problem is that:

*Feeding the constructed phonological word representation into the comprehension system for internal monitoring may yield a thread of selected nodes representing the internally perceived word and hearing self-produced overt speech may yield a thread of selected nodes in the comprehension system representing the externally perceived word. If the internally and externally perceived words are represented by different processing threads, the comprehension system can distinguish between them and prevent interference*. (2020, p. 15)

The question, however, remains: *How* does the system distinguish between them? Roelofs responds by appealing to condition-action rules. But how? Earlier I pointed out the problems with defining such rules for monitoring per se, and the problem of assuming that speaking, as an action, has a distinct “monitoring” goal (i.e., on top of the communication goal). A more practical solution stems from neural evidence of auditory suppression during self-produced speech (Aliu et al., 2008; Christoffels et al., 2011), which guarantees the quick suppression of the heard word. As described above, the HSFC takes advantage of this mechanism to simultaneously model internal monitoring and solve the cross-talk problem.

Given that a reasonable solution exists for the cross-talk problem in production-perception models, I will not dedicate extensive space to discussing the findings from the phoneme monitoring and eye-tracking tasks. But it is worth mentioning that the basic findings of Wheeldon and Levelt (1995), contrary to Roelofs’s claims, do lend empirical support to Vigliocco and Hartsuiker’s (2002) concern. The point relevant to this discussion is not whether the serial position effect is preserved or not. Phoneme monitoring is an attentional search task, and the empirical findings (e.g., Özdemir, Roelofs, & Levelt, 2007; Wheeldon & Levelt, 1995) provide convincing evidence that this search is sequential. So whatever else is changed, the nature of this process is not expected to change. Rather, the point relevant to Vigliocco and Hartsuiker’s (2002) criticism is whether concurrent production interferes with comprehension monitoring. It does: adding a concurrent articulation task delayed the monitoring of the first syllable onset by an average of 62 ms (Wheeldon & Levelt, 1995), and this was under circumstances that the verbalized sequence was highly repetitive and required no additional operations. It is thus not unreasonable to argue that the act of production should interfere substantially with the workings of a comprehension-based monitor.

Roelofs also dismisses differential timelines of looks to the cohort competitor (e.g., beaker/beaver) while producing speech and listening to other people’s speech (Huettig & Hartsuiker, 2010) as having implications for monitoring because the cohort word was not relevant to the picture naming task, so participants did not have a reason to fixate the words while planning the name. If this argument holds, then participants *never* have a reason to look at the cohort word, so a cohort advantage over unrelated items should never be observed in picture naming, but it was indeed present after naming the picture. The interpretation of Huettig and Hartsuiker (2010), in line with all interpretations of the competitor fixation effects that I know of, is that fixating the competitor is a non-deliberate action, sometimes in direct opposition to the task goal (e.g., Nozari, Trueswell, & Thompson-Schill, 2016).

### Evidence from aphasia

I agree, in principle, with Roelofs on the unreliability of correlational data. Issues of statistical power, task suitability, and coding make the interpretation of the results of correlational studies difficult. Moreover, the presence or absence of a correlation is also dependent on the details of the theoretical framework. For example, generally speaking, conflict-based monitoring predicts a relationship between the quality of the production system and the quality of monitoring (see Nozari et al., 2011, for simulations and detailed explanations of this relationship). However, such a relationship is expected based on the assumption that speakers would like to minimize the rate of false alarms, i.e., detecting their correct responses as errors. This preference, however, need not be absolute. Thus a simple shift in the criterion can change the expected relationship between production and monitoring (see Nozari & Hepner, 2018, for details). A similar general decision making framework is also applicable to signals from other kinds of monitor that do not operate with conscious explicit comparison between the target and response, and instead use some heuristic, e.g., the amount of residual activation in the percept as in HSFC, to detect the probability of an error. Therefore, while positive correlations are evidence in support of a theory, the absence of such correlations is not conclusive evidence against the theory.

Keeping these caveats in mind, we have now demonstrated the expected relationship between production and monitoring of lexical errors across 29 individuals with aphasia (Nozari et al., 2011), 62 children (Hanley, Cortis, Budd, & Nozari, 2016), and 20 English-Spanish bilinguals speaking their second language (Nozari et al., 2019). Roelofs questioned the validity of taking such correlations as evidence for the production-based monitor, arguing that the error detection data were based on overt rejection of a produced response, and thus could have come from either the internal or the external monitoring channel. This issue is addressed in Figure 4 of Nozari et al. (2011). The proportion of detected errors of each type (semantic vs. phonological) *only* shows a correlation with the strength of the connections related to that part of the production system, as predicted by a layer-specific production-based monitoring mechanism. This means that the probability that a semantic error will be detected by an individual with aphasia could be predicted from the estimated strength of the connections between semantic features and lexical items, but not from the strength of the connection in another part of the production system, or, for that matter, from comprehension scores. Figure 2 of Hanley et al. (2016) replicates this finding with semantic errors in children. The double dissociation between the detection of the two error types in relation to the two parts of the production system, and its dissociation from comprehension abilities, is incompatible with a monitoring channel that only has access to the final outcome of production, including the external monitoring channel.

Moving on from correlational data, arguments have also been made about the links between clinical aphasic syndromes and monitoring theories. I will address two such arguments below. One of the common arguments for linking monitoring to comprehension is the belief that Wernicke’s aphasics have monitoring problems, but Broca’s aphasics do not. While it is true that Wernicke’s aphasics often have poorer comprehension than Broca’s aphasics, it is often overlooked that their word production also shows signs of more extreme aberrations compared to Broca’s and Anomic aphasics, most likely because fluent production in individuals with Wernicke’s aphasia generates the impression of “better” production abilities. In reality, individuals with Broca’s and Anomic aphasia often produce errors that bear some relation to the target (e.g., “rat”, “dog”, or “cap” for the target “cat”). The neologisms in Wernicke’s aphasia, on the other hand, may bear no resemblance to the target and are often not real words (“firple” for the target “cat”). In computational terms, neologisms represent pure randomness in the production system, while other error types preserve some degree of systematic mapping (e.g., Dell, Schwartz, Martin, Saffran, & Gagnon, 1997). Thus, the two groups do not just differ in their comprehension abilities, but also in their production abilities, although because of the very different production profiles quantifying the level of production deficit across the groups is difficult. Furthermore, systematic studies of monitoring in individuals with Broca’s and Anomic aphasia show that such individuals do have pronounced problems in monitoring (e.g., Nozari et al., 2011; Schuchard et al., 2017). Therefore, the differences between Wernicke’s and Broca’s aphasics do not provide a watertight argument in favor of comprehension-based monitors.

Another group of individuals with aphasia, namely conduction aphasics, have also often been referred to in the monitoring debate. The common description of these individuals’ monitoring behavior is that their repeated attempts at repair bring them closer to the target. Often overlooked, however, is the fact that in many cases, they do not recognize a target when they hit it. For example, Kohn (1984) reports an individual who continues to repair his speech after he has correctly produced the target “igloo”: “/aj-, aj-, ajk-, ajgpl, ajpg-, ajglu, ej, iglu, ajglu, rgglu, glu, o, ajglu, Ijglu, li-, gli-, ajglu/, **igloo,** /iglu/, igloo…”. This behavior is a great example of quick successive repairs without explicit knowledge of the correct target, and as such is not a convincing piece of evidence for comprehension-based monitoring, which uses a clear standard of comparison, although it is compatible with both production-perception and production-based monitors.

### Error awareness, attention, and the ERN

The presence of ERN in cases of errors that were not consciously detected has been taken by us (and others) to provide support for monitoring mechanisms that could operate independent of conscious awareness. This, in turn, has been used to argue against the comprehension-based monitors which, as explained earlier, rely on conscious deliberate comparisons between a target and a response. Roelofs (2020) contests this claim by appealing to a meta-analysis concluding that the magnitude of the ERN can differ based on conscious awareness (Wessel, 2012). This conclusion, however, does not alter the original claim or its ramifications: the ERN is uncovered *even* on trials without conscious awareness, a finding that calls for a subconscious mechanism as its source. In interpreting the issues of statistical power, it is helpful to point out that the greater similarity in the amplitude of ERN in aware and unaware trials has been made in comparison with the significant differences observed between the two conditions on a later component, Pe, on the *same set of trials* (e.g., Ednrass et al., 2007). Note also that the direction of causality between the ERN and conscious awareness is unclear. It is quite possible that whatever mechanism generates the ERN subsequently triggers awareness. In such a case, it is reasonable to assume that larger ERN amplitudes (which mark stronger involvement of the underlying mechanism) would be more likely to trigger conscious awareness. The critical point remains that the absence of conscious awareness does not preclude the elicitation of the ERN on error trials.

A general note about subconscious processing and attention is useful here. Proposing that a process is implicit and largely subconscious does not imply that it is immune to attentional regulation (see Moors & De Houwer, 2006, for an extension to automatic vs. controlled processing). An example is speech segmentation by statistical learning. The underlying process is largely implicit (infants do not explicitly learn the phonotactic rules of the language they hear), but can suffer when attention is divided (Toro, Sinnett, & Soto-Faraco, 2005). These findings are not contradictory; they simply imply that sufficient activation of the underlying representations is vital, even for operations that do not entail the application of explicit rules. Attention, defined most simply as neural gain, guarantees the sufficiency of such activation in the neural population involved in a given task.

The idea of implicit subconscious monitoring and repair processes in language production supplemented by attentional control is gaining more weight. On the one hand, several pieces of evidence point to a basic fast subconscious process: children can repair their errors from an early age without being able to explain why they have changed their original utterance (Clark, 1978; Karmiloff-Smith, 1986). Rapid, repetitive repairs in individuals with aphasia, including both conduite d’approche for phonological errors, and semantic errors (Nozari, 2019) which continue past the production of the correct target, are other examples. Recently, in a single-word typing-to-dictation task, we asked participants whether they made an error/repair. Now replicated in three experiments with slight variations in the questions, we found clear evidence that in 10–20% of cases where an error was corrected (with the use of backspace + a new letter) participants had no awareness that an error had even occurred. When immediate visual feedback was removed (so that the outcome of typing was not immediately visible to the participants), we found the ERN, time-locked to each keystroke, for both consciously detected and undetected errors. The late positivity (Pe) which is the classic index of conscious awareness of errors, was, however, only present for the consciously detected errors (Pinet & Nozari, in press). These data suggest that conscious awareness is not a prerequisite for detecting a response as an error or for initiating a repair. On the other hand, participants have been reported to repair a higher proportion of their errors under more error-prone situations (Levelt, 1983; Nozari et al., 2019), and when accuracy is emphasized (Postma & Kolk, 1992). Collectively, these findings have led to proposals of an implicit monitoring/repair process which can be augmented by attention (Nooteboom & Quené, 2017; Nozari et al., 2019), but the data are incompatible with a monitoring mechanism that is fundamentally dependent on a conscious, deliberate mechanism for its operation.

### Anterior cingulate cortex (ACC) and performance monitoring

Very little is known about the neural correlates of monitoring, but one area that is very likely to be involved is the ACC (Gauvin, De Baene, Brass, & Hartsuiker, 2016; Riès, Janssen, Dufau, Alario, & Burle, 2011), an area that has been implicated in domain-general monitoring in a very large number of studies that do not involved producing language. Even in the non-linguistic cognitive control literature, the role of the ACC is still hotly debated, which is hardly surprising given the inhomogeneity of the ACC, hence the likelihood that it is a multi-functional structure (Vogt, Finch, & Olson, 1992). Excellent reviews of this debate exist elsewhere (e.g., Ullsperger, Fischer, Nigbur, & Endrass, 2014), so I will restrict the discussion here to how the neural data can constrain monitoring theories in language production.

The first point concerns Roelofs’s claim that the congruency sequence effect (CSE), i.e., adjustments to performance after encountering congruent and incongruent trials, which has been linked to the ACC, results from expectation and not conflict monitoring. In a clever series of experiments, Jiménez and Méndez (2013) tested the predictions of these two accounts by looking at the CSE after a sequence of either congruent or incongruent trials. They found the largest CSE after a long sequence of congruent (low conflict) trials, even though participants had clearly indicated that they had expected a switch to an incongruent trial at that point. Interestingly, while participants also indicated their expectation for a switch after a long series of incongruent trials, the CSE was reduced after a series of congruent trials. In other words, a dissociation was found between expectations and the CSE. The direction of the CSE change, however, was in line with the predictions of the conflict-based account: a long series of low-conflict trials decreased the amount of control, leading to a larger CSE, whereas repeated encounters with high-conflict situations increased the amount of control, leading to a smaller CSE. The findings of Jiménez and Méndez (2013) highlight the importance of subconscious, cumulative adjustments to performance (see also Freund & Nozari, 2018, for an incremental learning account of CSE along the same lines) that can be dissociated from explicit knowledge and expectations.

The second point concerns Roelofs’s criticism of the view of ERN/N2—whose origins have been traced back to the ACC—as indices of conflict and behavior. A similar criticism has been brought up in a recent study by Zheng, Roelofs, Farquhar, and Lemhöfer (2018). ERN is much more extensively studied in forced-choice button-press tasks compared to language production tasks, so I start by addressing the findings of Burle, Roger, Allain, Vidal, and Hasbroucq (2008), which have been taken as conclusive evidence against a conflict monitoring account of the ERN. Three points are worth noting:

- The measure of conflict used is Hopfield energy. Calculated as –*∑∑a_i_a_j_w_ij_*, Hopfield energy is a function of the product of the activation of two response nodes (*a_i_* and *a_j_*, respectively) scaled by the weight of the inhibitory connection between them (*w_ij_*). A good measure of conflict must reliably differentiate between situations of high and low conflict. Imagine a case with the highest conflict (node 1 and node 2 both have exactly the exact same activation, say 0.3), and a case with very low conflict (node 1 has an activation of 0.1, while node 2 has an activation of 0.9). Multiplication of activations in both cases returns the same value of 0.09. Since the connection weight *w_ij_* is the same in both cases, conflict ends up being estimated as identical in these two very different situations. While Hopfield energy works well as a conflict measure in certain networks, it is not a reliable measure of conflict in all models, and is a particularly dangerous measure for evaluating the claims of the theory.
- For a model to be tested on a dataset, the dataset must meet the basic assumptions of the model. In case of Burle et al. (2008), the assumption has been that the electromyography activities pertaining to partial and complete responses overlap in time, and that the amount of such overlap could be correlated with the magnitude of the ERN. In practice, no such overlap was observed in the empirical dataset modeled by Burle et al. (2008). The authors could have simply concluded that the *empirical evidence*—not the model—is altogether incompatible with the concept of response conflict (and there is thus nothing to model). Note, however, that such overlap has previously been reported at the trial level (e.g., Carbonnell & Falkenstein, 2006), so it is unclear what factors have caused its absence in the study of Burle et al. (2008). Moreover, the conflict-based account is not the only account of the ERN which assumes the activation of multiple response alternatives at the motor level (e.g., Holroyd & Coles, 2002).
- Empirical issues aside, the authors assume that high temporal overlap between two motor responses represents the amount of conflict important for selection. If we unpack this, the amount of overlap translates to how quickly a final response was made after an incomplete response, with larger overlap meaning faster generation of the final response. In other words, the assumption is that, in two trials which both started with the partial incorrect response, the high-conflict trial lead to a faster final response compared to the low-conflict trial. No conflict-based monitor makes such a prediction. The relationship between the incomplete and complete responses can, however, be viewed in a different light. We could assume that the incomplete response marks the first selection attempt, which is then overturned by the generation of the complete response. The complete response must ultimately have higher activation to be selected. It is the amount of activation of the incomplete response that determines how long it takes for the complete response to override it. The higher the activation of the incomplete response, the longer it would take for the complete response to overtake it, hence a longer gap between the two. In keeping with this view, Fig. 5B in Burle et al. (2008) shows a parallel increase in the activation of the incomplete response and the temporal overlap between the two responses. The gap between the two responses is thus not a good proxy for the amount of conflict, but instead for the activation level of the incomplete response, which is not by itself indicative of the amount of conflict between the two responses.

**Figure 3:**
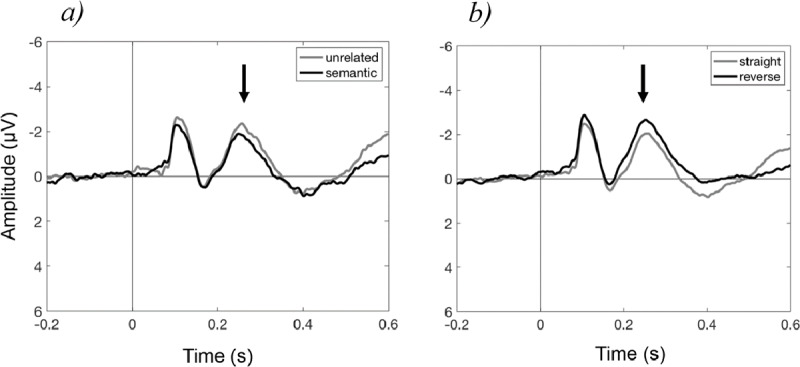
The N2 in language production (Cz is shown). **a)** Production of the target (e.g., “cat”) in the presence of an unrelated competitor (e.g., “pen”) vs. in the presence of a semantically related competitor (e.g., “dog”). **b)** Production of the target from the target picture vs. from the picture of a different item. In both cases, the black line represents the condition that led to behavioral interference, but this interference cannot be predicted from the direction of change to the N2.

For these three reasons, the results of Burle et al. (2008) do not provide conclusive evidence against general accounts of conflict monitoring. Let us now turn to the ERN and N2 in the context of language production. The biggest problem here is the paucity of data; very few studies have investigated ERN and N2 in language production (e.g., Costa et al., 2009; Ganushchak & Schiller, 2008a, b, 2009). In a recent review, we have discussed the findings of the existing studies, along with the unresolved discrepancies with the larger literature, and concluded that the evidence is simply not enough to attach a theoretical explanation to them (Nozari & Pinet, in press). Moreover, importing explanations from the non-linguistic literature, as in the case of N2, runs into problems. For example, Costa, Strijkers, Martin, and Thierry (2009) reported monotonically increasing RTs as more pictures with objects belonging to the same category were named, accompanied by a decrease in the amplitude of N2. In keeping with this, a recent study in our lab found lower N2 amplitudes for naming the same picture when the other picture in the block was a semantically related item, compared to an unrelated item (Figure 3a). Therefore, the condition that created more interference, and was associated with indices of greater behavioral difficulty, showed a *lower* N2 amplitude. When, however, the names of the two pictures in the block had to be swapped, i.e., speakers had to say “cat” upon seeing the picture of a “dog” and vice versa (Nozari et al., 2016), the amplitude of N2 was higher compared to when the same pictures were to be named by their canonical names (Figure 3b). Here, the condition that created greater interference, which we often interpret as requiring greater inhibitory control, showed a *higher* N2 amplitude. What can we conclude from these and similar results? In my opinion, that we simply do not yet have a good enough grasp on what ERP components signify in word production to attach theoretical interpretations to these components.

In light of this, and the general complexity of linking ERP components to behavior even for well-known components like the N400 (Cheyette & Plaut, 2017; Rabovsky & McRae, 2014), I am not sure how testable predictions can be derived without a solid model-driven approach that actually generates the ERP components and models their relationship with various experimental factors (e.g., see Cheyette & Plaut, 2017, for such an approach towards the modeling of the N400). Emphasizing that our behavioral conflict detection model does *not* make testable predictions regarding ERN or any other ERP components, I can say that in so far as an ERP signature is assumed to be a reliable index of error probability, some of the predictions attributed to the model are in fact not what the model would predict. For example, Zheng et al. (2018) replicated the well-known asymmetry in bilingual language switching, with more errors when switching from L2 to L1 (L2→L1) than vice versa. The ERN magnitude, however, was larger during the switch from L1 to L2 (L1→L2). These results were taken as evidence against conflict-based monitoring. Let us ignore the fact that language switching most likely induces conflict at a level different from lexical selection during monolingual production (Schuch, Dignath, Steinhauser, & Janczyk, 2019), and assume that there is one source of conflict and that it generates higher conflict in the L2→L1 switch than in the L1→L2 switch. This holds for *all* trials, regardless of whether they lead to an error or a correct response. This, in turn, implies that the same amount of conflict is less informative in L2→L1 than L1→L2 switches, simply because it cannot distinguish between error and correct trials nearly as reliably. Thus, to the extent that ERN is taken as an effective proxy for errors, the model, if anything, would predict weaker ERNs in L2→L1 than in L1→L2, as Zheng et al. (2018) have found. As I have pointed out before, however, establishing such a link requires a model of ERN generation in language production, which is yet to be proposed. Regardless of the specific model, the absolute value of conflict is of little importance in conflict-based models; what matters in generating a reliable error signal is the difference between the amount of conflict on correct and error trials.

In summary, the ERP components in language production and their link to behavior are not well understood. The little we know does not rule out production-based monitoring. If anything, they endorse some of the characteristics of production-based (and production-perception) monitors, such as subconscious monitoring, over comprehension-based monitors.

### Neuroimaging evidence

Some behavioral features, e.g., reliance on conscious awareness, can distinguish between some of the theoretical accounts of monitoring in language production. Disentangling production-based and production-perception monitors such as HSFC remains somewhat difficult though. Neuroimaging data provide an additional source of evidence for the evaluation of different theories of monitoring. It is thus important that the theory is well-formed enough to have clear biological correlates. The involvement of the medial and lateral prefrontal cortices in monitoring is undisputed (e.g., Gauvin et al., 2016; Riès et al., 2011), but, as pointed out by Roelofs (2020), can be accommodated by various theories, although different accounts attribute different functions to these regions.

On the other hand, a critical role for perceptual regions is only assumed by models that view perceptual representations as a key part of the language monitoring system. For example, HSFC includes two monitoring loops (Hickok, 2012): syllable-level monitoring involves the auditory cortex, the pre-motor and motor cortices, and area Spt (Sylvian parietal junction) as the coordinate transform zone. Monitoring at the level of articulatory feature clusters (which Hickok roughly equates with phoneme-level monitoring) involves the somatosensory cortex, lower primary motor cortex (M1), and cerebellum as the mediator between the two. Comprehension-based accounts, as far as I know, have not explicitly postulated a link between the models and neural regions corresponding to them. Monitoring via the external channel should have the same neural correlates as comprehension. Since monitoring via the internal channel has been described as monitoring inner speech, it is reasonable to assume that its neural correlates are those described for inner speech. These include left inferior frontal gyrus, dorsal premotor cortex, area Spt, posterior superior temporal sulcus, and superior temporal gyrus, or STG (Buchsbaum, Hickok, & Humphries, 2001; Buchsbaum, Olsen, Koch, & Berman, 2005; Hickok et al., 2003). Production-based monitors link production directly to monitoring abilities. Involvement of specific regions depends on the stage of production: at lower levels, i.e., closer to syllabification and motor production, the critical regions would be the same motor regions implicated in production-perception models. At higher levels, i.e., lexical selection, critical regions are the middle temporal gyrus and the inferior frontal gyrus, which, as hypothesized by conflict-based models, implements control over the temporal cortex in order to keep the amount of conflict low (e.g., Schnur et al., 2009). The role of the medial and dorsal surfaces of the prefrontal cortex are not to supervise monitoring, as claimed in comprehension-based accounts, but these regions are hypothesized to receive signals indicating the need for control and, in turn, to deploy such control towards the part of the system in need.

Neuroimaging studies of speech monitoring, especially of the internal channel, are rare, and blocking the external channel with noise has two problems: First, noise-masking cannot completely block the external channel because of bone conduction. Second, alteration of auditory feedback induces the Lombard reflex (Lane & Tranel, 1971), which changes the loudness and fundamental frequency of speech and slows down articulation. In other words, noise-masking changes the primary production processes. This is not ideal, but does not preclude a noise-masking design from providing valuable information about speech monitoring. An example is the study of Gauvin et al. (2016), in which the authors compared the neural correlates of error detection in tongue-twisters produced by others or by speakers themselves under noise-masking. Roelofs (2020) criticized this study by pointing out that the perception system is activated differently under the two conditions, so a direct comparison of its activity between production and perception is bound to yield a complex pattern. He then concluded that, contrary to the authors’ claims, the complicated way in which errors “activated” superior temporal cortex (as well as ACC and frontal regions) is compatible with the comprehension-based monitor.

It appears to me that some important points are lost here. For one thing, the critical comparisons in Gauvin et al.’s (2016) study included within-condition comparison of error vs. correct trials and the interactions of these comparisons with conditions, so neural activity was compared to the correct baseline, even when monitoring performance was compared for speech produced by self vs. others. More importantly, in none of the comparisons did errors “activate” the superior temporal cortex; quite the contrary, when a reliable difference was found, it was in the direction of *decreased* activity of the STG during error production than correct production. Without appealing to a specific model, Gauvin et al. (2016) predicted that comprehension-based monitoring should involve greater activation of the STG on error trials, which was contrary to what was found. As explained earlier, more detailed models of production-perception monitoring, e.g., HSFC, also predict greater activity in STG on erroneous trials. In light of this, it is hard to interpret Gauvin et al.’s results as compatible with comprehension-based monitoring.

## Towards a complete model of monitoring in language production: one or multiple mechanisms?

I reviewed three main classes of monitoring models that have been proposed for monitoring self-produced speech. Each class has unique strengths, which also define constraints on the scope and mechanism of the model. For example, a comprehension-based model, through its use of the parser, allows for a sophisticated analysis of syntactic structures, as well as the pragmatics of language. This kind of monitoring, by definition, draws on the speaker’s linguistic and social knowledge, and cannot operate without conscious awareness. Production-perception models such as DIVA and HSFC are powerful models for explaining how speech is learned and controlled at the pre-motor and motor level, but they operate on distinct sets of representations on the production and perception sides, which limits their utility in higher levels of the language system. Production-based monitors (and their predictive temporal variants) are readily applicable to higher levels of the production system, and can operate subconsciously and without a “standard of correctness”, which makes it possible for them to detect an error without any specific knowledge about the target.

Which model is the right model? Let me present four arguments for why, I believe, this is the wrong question.

1. Unlike the motor speech tradition of language production, the psycholinguistic view maintains that the language production challenge involves a host of higher level processes, starting from mapping a very complex semantic space to lexical items. Both the nature of representations and the dynamics of mapping are very different at these levels, compared to lower-level mapping of a speech motor plan to articulatory phonetic features. Most prominently, semantic-lexical mapping is context-dependent and non-deterministic. It is well-established, both by behavioral and neural evidence, that even simple concepts such as “lemon” are not stable across individuals and contexts (see Yee & Thompson-Schill, 2016 for a review). Moreover, the same concept may map onto different lexical items, e.g., the concept of “not giving in to pressure” may evoke the words “resistance”, “resilience”, “patience”, etc., the choice of which depends on the broader context. The same word also may be polysemous, i.e., it may have multiple meanings. In other words, the nature of semantic-lexical mapping is “many:many”. This is generally not true for mappings at the lower levels of the production system. Once a lexical item has been chosen, the mapping of each motor plan to its articulatory phonetic features is more or less deterministic. Moreover, there is a finite (and quite limited) number of such features in a given language, which is quite different from the great diversity of concepts and lexical items. In short, the two parts of the system must work with very different kinds of constraints. One deals with a vast number of flexible representations and requires versatility and consideration of the greater context. The other deals with a much smaller set of representations and a much less variable mapping, save for co-articulation adjustments. They have different monitoring needs, which I argue, are best accommodated by monitoring mechanisms that are closely coupled with the nature of the representations and dynamics of mapping at each stage.
2. In a sense, the argument for multiple monitoring systems has already been demonstrated in the division of labor between internal and external monitoring channels (Hartsuiker & Kolk, 2001; Lackner & Tuller, 1979; Nooteboom, Quené, 2017; Pinet & Nozari, in press). Some aspects of speech simply rely on auditory information for monitoring, such that blocking auditory feedback changes the pitch and rate of speech (Lane & Tranel, 1971). At the same time, other aspects of speech are much more resilient to blocking, such as the detection of semantic errors (see Hartsuiker & Kolk, 2001, and references therein). A similar dissociation is observed in consequences of monitoring: we have recently shown that blocking external feedback (visual word in typing) has little effect on error awareness, but a tremendous effect on repair attempts. Moreover, we have demonstrated that the electrophysiological signatures of monitoring with and without overt feedback show non-negligible differences (Pinet & Nozari, in press). It is thus hard to argue that it is the same mechanism that operates both internally and externally (see my other arguments against this in the body of the paper). These findings and many others point to distinct, albeit complementary, monitoring mechanisms that push the argument for a multi-component monitoring system from the theoretical to the empirical realm.
3. Objections to a multi-component monitoring system may be raised on grounds of parsimony; it feels like an overkill to have several monitoring mechanisms just for language production. A quick look at other biological systems for monitoring suggest otherwise. A good example is monitoring balance. There are three distinct mechanisms for balance monitoring in human body, through visual input, through the vestibular system, and through the proprioceptive system. Given that standing and walking are highly practiced behaviors, and arguably much less complex and generative than a task such as language production, it is not unreasonable to argue for similar redundancies in language monitoring. In fact, even different auditory vs. proprioceptive routes have already been proposed in Guenther and Hickok’s models, within the domain of motor control, which, as argued above is not the only component of the language production system. With scaling up to higher levels, the idea of a multi-component monitoring system only gains more strength.
4. To the above, I add the need for monitoring for more complex aspects of communication, e.g., making sure the interlocutor is following the conversation by adopting their theory of mind, as well as the referential communication context. The latter, also discussed in Levelt (1983) as appropriateness repairs, requires deliberation, attention to common ground, history of the conversation, and various factors related to the interlocutor (age, social situation, cognitive status, etc.). This aspect of monitoring, and the subsequent repairs, is certainly far from subconscious and “automatic” (e.g., Trude & Nozari, 2017), as has been argued for other aspects of monitoring such as quick detection and replacement of errors with repairs (Nooteboom & Quené, 2017; Nozari et al., 2019), providing yet another reason for why a multi-component monitoring system is the right direction for arriving at a comprehensive model of monitoring in language production.

Let me end by pointing out that having multiple mechanisms for monitoring production does not preclude a common framework for integrating the contribution of those mechanisms. We have recently argued that a more general notion of conflict (not limited to that used by Nozari et al., 2011, in production-based monitoring) provides a viable framework for such discussions (Nozari & Hepner, 2019a, b; see also Gauvin & Hartsuiker, this issue). Specifically, we have shown that regardless of the specific mechanism, all models of monitoring use variables and comparisons that can be quantified by the notion of conflict (as capturing information about the difference between the activation of two representations), and critically, all such models are aligned in their predictions that higher conflict is associated with a higher probability of errors (Pinet & Nozari, under review). The use of conflict as common currency across different accounts allows for quantifying the contributions of various mechanisms to different functions (e.g., metacognitive judgments vs. correction behavior), without forcing a single mechanism on monitoring at all levels of production and for different aspects of communication.

## Conclusion

Although this paper addresses the arguments raised by Roelofs (2020) in his defense of comprehension-based monitoring, I took this opportunity to argue for a different point of view that encompasses a single monitoring mechanism. I reviewed several mechanisms in detail and pointed out the strengths and limitations of each. I also discussed two temporal models with operations that resemble “prediction”. Finally, I laid out arguments for why picking only one is neither theoretically, nor empirically, the most appropriate choice. Instead, I believe that a more fruitful approach would be to investigate which monitoring mechanism is capable of providing useful and timely information for a given aspect of production, how information from multiple monitoring mechanisms are combined, and the extent to which one mechanism can compensate for the loss of another.
